# Oxazolone-Induced Immune Response in Atopic Dermatitis Using a Goat Model and Exploration of the Therapeutic Potential of Pomegranate Peel Extract

**DOI:** 10.3390/ani15030411

**Published:** 2025-02-02

**Authors:** Ahmed Elfadadny, Haney Samir, Ahmed S. Mandour, Rokaia F. Ragab, Besheer G. Elshafey, Fawaz E. Alanazi, Helal F. Hetta, Ahmad A. Alharbi, Abdullah S. Albalawi, Suhailah S. Aljameel, Maha Abdullah Alwaili, Wedad M. Nageeb, Mahmoud H. Emam

**Affiliations:** 1 Laboratory of Internal Medicine, Cooperative Division of Veterinary Sciences, Graduate School of Agriculture, Tokyo University of Agriculture and Technology, Fuchu 183-0054, Japan; 2 Department of Animal Internal Medicine, Faculty of Veterinary Medicine, Damanhour University, Damanhour 22511, Egypt; besheer_elshafey@vetmed.dmu.edu.eg; 3 Department of Theriogenology, Faculty of Veterinary Medicine, Cairo University, Giza 1221, Egypt; haneyvet360@yahoo.com; 4 Laboratory of Veterinary Physiology, Department of Veterinary Medicine, Faculty of Agriculture, Tokyo University of Agriculture and Technology, Fuchu 183-0054, Japan; 5 Department of Animal Medicine (Internal Medicine), Faculty of Veterinary Medicine, Suez Canal University, Ismailia 41522, Egypt; dr_mandour@vet.suez.edu.eg; 6 Department of Biochemistry, Faculty of Veterinary Medicine, Damanhour University, Damanhour 22511, Egypt; 7 Department of Pharmacology and Toxicology, Faculty of Pharmacy, University of Tabuk, Tabuk 71491, Saudi Arabia; falnazi@ut.edu.sa; 8 Division of Microbiology, Immunology and Biotechnology, Department of Natural Products and Alternative Medicine, Faculty of Pharmacy, University of Tabuk, Tabuk 71491, Saudi Arabia; hhussen@ut.edu.sa (H.F.H.); aam_alharbi@ut.edu.sa (A.A.A.); 9 Department of Pharmaceutical Chemistry, Faculty of Pharmacy, University of Tabuk, Tabuk 71491, Saudi Arabia; abs_albalawi@ut.edu.sa; 10 Department of Chemistry, College of Science, Imam Abdulrahman Bin Faisal University, Dammam 31441, Saudi Arabia; ssaljameel@iau.edu.sa; 11 Department of Biology, College of Science, Princess Nourah Bint Abdulrahman University, P.O. Box 84428, Riyadh 11671, Saudi Arabia; maalwaele@pnu.edu.sa; 12 Department of Medical Microbiology and Immunology, Faculty of Medicine, Suez Canal University, Ismailia 41522, Egypt; wedad_saleh@med.suez.edu.eg; 13 Department of Animal Medicine, Zagazig University, Zagazig 44511, Egypt; mh5378721@gmail.com

**Keywords:** atopic dermatitis, goat, Th2-driven inflammation, PPE

## Abstract

Atopic dermatitis (AD) remains a challenging condition, necessitating effective models for investigation and therapeutic exploration. In this study, we employed a goat skin model to assess the immune response triggered by topical oxazolone (OX) application. The OX simulates a Th2-driven immune reaction typified by elevated Th2 cytokine (IL-4 and IL-13) expression and the infiltration of mononuclear cells magnified by the presence of CD3^+^ and CD4^+^ T-cells in the epidermal and dermal layers. Additionally, the application of pomegranate peel extract (PPE) demonstrated a mitigating effect on OX-induced skin alterations, suggesting potential therapeutic benefits. This study underscores the value of goats as a reliable and accessible model for studying AD in humans and/or large animals. While the therapeutic potential of PPE dosage requires further exploration.

## Simple Summary

The expression of cytokines in livestock remains largely unexplored. This study aimed to investigate cytokine expression in a goat model of atopic dermatitis (AD). By establishing this novel goat model, we aimed to more closely replicate the pathophysiology of dermatitis in humans and larger animals, offering a more relevant alternative to traditional mouse models. The goat model, being more similar in size and physiology to large animals and humans, exhibited cytokine expression patterns akin to those seen in previous mouse studies, as confirmed by histopathological analysis and immunostaining. These preliminary findings open new avenues for further research on AD in large animals and humans, underscoring the potential of the goat model for broader dermatological studies.

## 1. Introduction

The skin serves as the largest organ in the animal body, playing a crucial role in overall animal health. It consists of two main layers: the epidermis and the dermis [1]. The epidermis is made up of layers of keratinocytes at various stages of differentiation. Functionally, the epidermis acts as a barrier, safeguarding the body from external factors such as physical stimuli and ultraviolet light. The goat holds significant importance in the livestock industry, meeting various human needs—economic, cultural, agricultural, and even religious—since the early stages of human civilization [2,3]. The health of a goat’s skin emerges as a critical factor influencing optimal productivity and, consequently, the profitability of livestock farmers. The inflammatory reactions in a goat’s skin in response to diseases are diverse, influenced by the specific agent, the cellular level of skin involvement, and the affected skin’s location.

Atopic dermatitis (AD) is an inflammatory skin condition linked to diverse factors, including immunological abnormalities and exposure to allergens. It presents with symptoms such as itching, eczema, and susceptibility to pathogenic infections [4]. The inflammatory response in AD involves T helper (Th) cells, which play a pivotal role. These cells are categorized as Th1, Th2, or the recently identified Th17 cells, based on the specific cytokines they produce [5]. This classification depends on the type of immune response activated in the skin, contributing to the pathogenesis of AD [6]. The initiation of the Th1 cascade is characterized by the production of pro-inflammatory cytokines such as IL-1, IL-2, IFN-γ, and TNF-α. This activation subsequently triggers neutrophils and macrophages, leading to cell-mediated immunity. In contrast, the Th2 immune response is independent of phagocytes and is characterized by robust antibody responses, along with the accumulation of eosinophils and basophils. Key cytokines associated with the Th2 response include IL-4, IL-5, IL-6, IL-9, IL-10, and IL-13. Th17 cells, a distinct lineage of helper T cells identified in the last two decades, are characterized by their production of IL-17A [7]. These cells play a crucial role in defending against various pathogens and have been implicated in the pathogenesis of numerous inflammatory and autoimmune diseases.

*Punica granatum* L., commonly known as pomegranate, belongs to the *Lythraceae family* and is a deciduous tree found worldwide [8]. The pomegranate peel extract (PPE) is recognized for its significant nutritive value, health-promoting attributes, and the presence of antioxidant bioactive compounds. PPE has been extensively utilized in herbal medicine, particularly in addressing various health issues such as the flu and upper respiratory tract infections [9,10]. Interestingly, all parts of the pomegranate fruit, including the peel and seeds, often considered by-products, can be processed to create value-added products with applications in industries, medicine, and cosmetics. PPE exhibits anti-inflammatory properties by inhibiting both lipoxygenase and cyclooxygenase enzymes and by reducing the levels of inflammatory cytokines [10,11].

The oxazolone-induced contact dermatitis model in murine studies has shown potential in mimicking Th2 cytokine-associated dermatitis observed in dogs. This model relies on repeated application of oxazolone to skin tissue, which sensitizes it and, upon re-exposure, recruits effector T cells. This process induces inflammation mediated primarily by CD3^+^, CD4^+^, and CD34^+^ T cells, closely resembling the immune and inflammatory features characteristic of canine atopic dermatitis [12,13].

However, the immune response to OX in other species, such as goat skin, remains unclear. Given the increasing acceptance of goats as a model for biomedical research and surgical training over many decades, our study aims to understand how goats’ skin responds to OX application and to explore the therapeutic potential of PPE as a promising agent to alleviate the symptoms of AD in goat skin.

## 2. Materials and Methods

### 2.1. Chemicals

Oxazolone (4-Ethoxymethylene-2-phenyl-2-oxazolin-5-one, 862207-1G-A, CAS Number: 15646-46-5) was purchased from Sigma-Aldrich (St. Louis, MO, USA).

### 2.2. PPE Preparation

The preparation of pomegranate peel extract followed the method outlined by Dahham et al., utilizing the methanol extraction technique [14]. In brief, fine peel powders were obtained through an electric blender and subjected to oven-drying at 40 °C for 24 h. Subsequently, the powders were filtered through a 24-mesh sieve, and 10 g of the resulting powder sample were extracted with 250 mL of 80% methanol at room temperature for 24 h. The resulting extract underwent filtration and was then diluted in a water/glycerin mixture (2:1) for optimal suitability for topical application on the skin.

### 2.3. Animals

In the current study, twelve sexually mature female Shiba goats (*Capra hircus*) were employed, with an average age of 24.7 ± 2.1 months and a body weight of 27.4 ± 4.5 kg. These goats were housed in a paddock under natural daylight conditions. Shiba goats, are known as Japanese miniature nonseasonal breeder goats. The goats were maintained on a diet comprising hay cubes (400 g per animal twice a day), with access to mineralized salt licks and water provided ad libitum. The goats used in this study were housed at the Veterinary Medicine Department, Faculty of Agriculture, Tokyo University of Agriculture and Technology, in Fuchu, Tokyo, Japan (Latitude 35.67° N; Longitude 139.48° E). They were maintained under standard management conditions, with a controlled temperature of 20–25 °C and relative humidity of 35–40%. The goats were provided with a balanced diet comprising concentrates and roughages to meet their nutritional needs, with free access to water and mineral blocks. Housing facilities were spacious, allowing ample room for movement and relaxation, and bedding was inspected daily to ensure cleanliness and replaced as needed. Prior to the experiment, all goats were clinically healthy, exhibiting no signs of disease or skin issues. All procedures were conducted in compliance with the ethical guidelines for animal use established by the Tokyo University of Agriculture and Technology, Japan (Ethical Approval Number: 30-78).

### 2.4. Induction of Model of Goat AD

**Figure 1:**
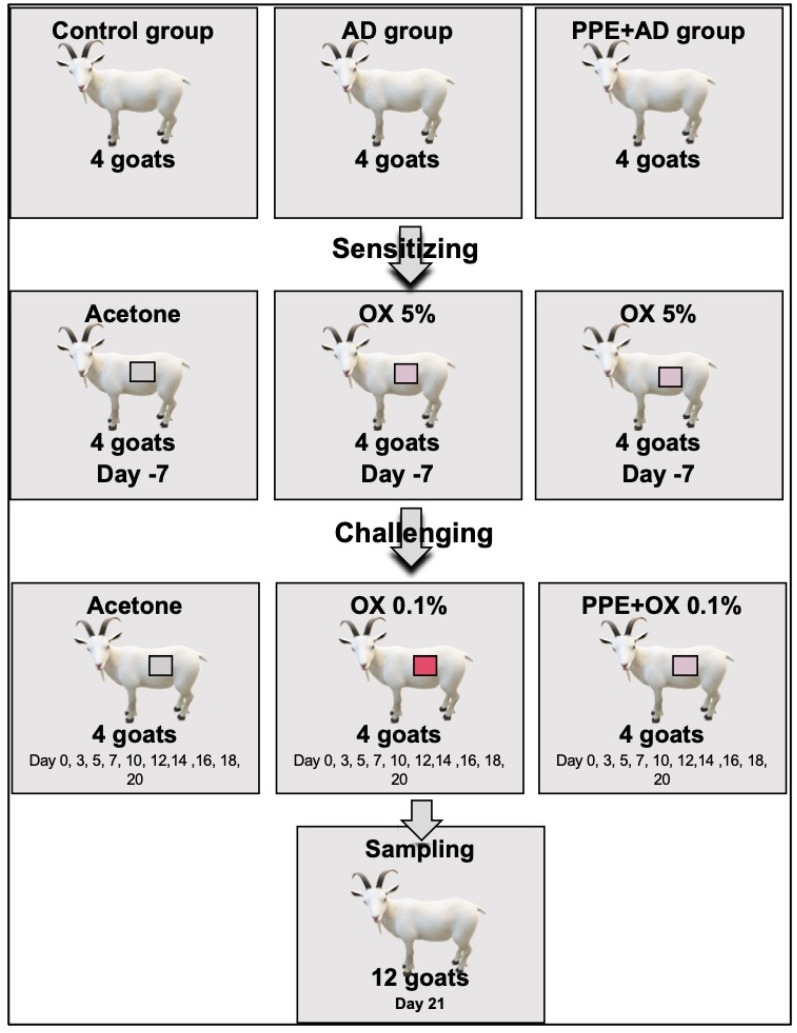
Experimental design overview. Twelve goats were divided into three groups: control, AD, and PPE + AD. Sensitization involved the application of 5% oxazolone, followed by a 3-week challenge period with 0.1% oxazolone at 2-day intervals.

In order to establish an AD model in goats, the animals were categorized into three experimental groups, each consisting of four goats (*n* = 4 per group). On the lateral part of the left abdominal wall, the groups received distinct treatments, namely the control group, AD group, and PPE + AD group. The AD and PPE + AD groups underwent sensitization through a single topical application of 5% OX in acetone (80 μL/2 cm^2^), while the control group was sensitized with an equivalent amount of acetone. Following one week of sensitization, the AD and PPE + AD groups were subjected to topical challenges with 0.1% OX at the same site at 2–3 day intervals for a duration of 3 weeks. In the PPE + AD group exclusively, and preceding each challenge, 80 μL of 20% PPE diluted in water/glycerin was applied to the goat skin prior to the application of OX, Figure 1.

### 2.5. Evaluation of Skin Lesions

The assessment of the severity of lesions on the specified area of the goat skin utilized a modified scoring system adapted from the AD scoring system commonly employed for dogs [15,16]. The examination focused on key indicators, including erythema, edema, itching/local alopecia, and excoriation with the formation of crusts. To quantify the severity, total skin severity scores were defined as the sum of individual scores (0, no signs; 1, mild; 2, moderate; 3, severe) for each of the four previous signs. Additionally, the skin thickness of each goat was measured using a manual skin caliper. During the measurement, the goats were positioned squarely on an even surface to ensure stability. The skin fold was then carefully measured in millimeters, providing an objective and consistent metric to assess changes in skin structure and thickness throughout this study.

### 2.6. Histopathological Examination

Skin samples were obtained using an 8 mm disposable biopsy punch (Kai industries Co., Ltd. 1110 Oyana, Seki-shi, Gifu, 501-3992, Japan) 24 h after the last application. Each harvested sample was divided into two halves: the first half was promptly stored in RNAlater for subsequent real-time qRT-PCR analysis, as discussed later, while the second half was fixed in 10% neutral buffered formaldehyde solution for 36 h for histopathology. The fixed tissue sections were embedded in paraffin wax and sliced at a thickness of 5 μm. These sections were then stained with hematoxylin and eosin (H&E) to assess histological changes and inflammatory cell infiltration in the three experimental groups. The images were examined under an optical microscope (BX43F, Olympus; Tokyo, Japan) and captured using a digital camera (DP73; Olympus). Epidermal thickness was calculated using image software (CellSens Standard V1.16; Olympus, Tokyo, Japan) among the three groups.

### 2.7. Immunohistochemistry

For the immunohistochemistry assay, paraffin-embedded tissues underwent sectioning and deparaffinization using xylene and graded alcohol. Antigen retrieval was carried out by incubating slides for 15 min at 95 °C in citrate buffer (pH 6.0). To block the endogenous peroxidase reaction, slides were treated with 3% H_2_O_2_ for 15 min and subsequently blocked with 5% goat serum, which was diluted in PBS with 0.1% Tween^®^ 20 (Fujifilm Wako Pure Chemical Co., Osaka, Japan). Sections were then incubated overnight at 4 °C with the primary antibodies listed in Table 1. Following incubation, slides were washed with PBS (3 times × 5 min) and incubated with horseradish peroxidase-conjugated secondary antibody IgG (H + L) for 1 h at room temperature. After a second washing step, antibody localization was visualized using 3,3-diaminobenzidine with hematoxylin counterstaining, and the stained sections were observed under an Olympus microscope (BX43F, CellSens Standard; Olympus; Tokyo, Japan). Images were captured using a digital camera (DP73; Olympus). For statistical analysis, two sections per skin biopsy/goat were sliced, resulting in a total of 8 sections per group (2 sections × 4 goats = 8 sections per group, totaling 24 sections/all groups). The average number of CD3^+^, CD4^+^ T cells, and CD34 intensity was calculated per section, and the cell numbers are expressed as cells/mm^2^.

### 2.8. Real-Time qRT-PCR

To assess the expression of Th1 and Th2 cytokines in the three goat groups, skin tissue was collected and preserved in RNAlater solution (Sigma-Aldrich, St. Louis, MO, USA) until processing. The samples were homogenized using a prechilled bead crusher (Bead Crusher μT-01, TAITEC, Saitama, Japan). Total RNA extraction was performed using the Illustra RNAspin Mini RNA isolation Kit (GE Healthcare, Chicago, IL, USA) following the manufacturer’s protocol. RNA concentration was determined, and samples were stored at −80 °C for subsequent analysis by RT-PCR. For cDNA synthesis, 1 μg of RNA was utilized with the primescriptTM RT master mix (perfect real-time) from Takara kits (Takara Bio Inc., Kusatsu, Japan). Primers for gene expression were designed and are listed in Table 2. Real-time qRT-PCR was carried out using the Thermal Cycler Dice Real Time System II (Takara Bio Inc.) and TB green premix EX Taq II (Tli RNase H plus) under the following conditions: 5 min at 95 °C, 40 cycles of 10 s at 95 °C, and 30 s at 60 °C followed by a dissociation cycle. Expression levels of target genes were normalized to two housekeeping genes, GAPDH and β-Actin. The comparative relative expression ratio was calculated using the 2^−ΔΔCt^ method, where ΔΔCt = ΔCt_sample_ − ΔCt_calibrator_.

### 2.9. Statistical Analysis

The statistical analysis was performed using GraphPad Prism7 version 7.01 (GraphPad Software, Inc., San Diego, CA, USA). The Mann–Whitney U-test was applied to evaluate differences in epidermal thickness, comparing the control group with both the AD group and the PPE + AD group. Similarly, this test was employed to identify significant differences in the frequency of CD3^+^ and CD4^+^ T cells among the three experimental groups. Furthermore, the Mann–Whitney U-test was utilized for the analysis of Th1, Th2, and Th17 cytokine expression. A *p*-value less than 0.05 was considered statistically significant.

## 3. Results

### 3.1. Clinical Assessment of AD Model in Goat

**Figure 2:**
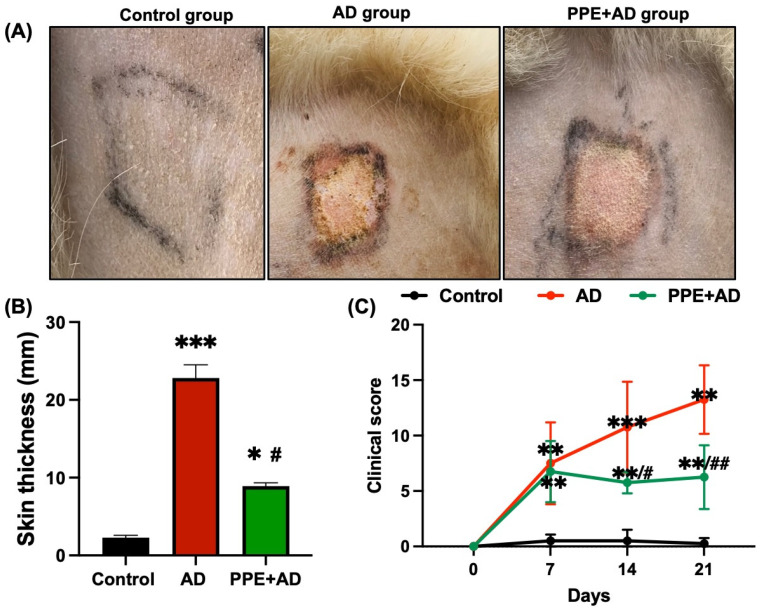
Induction of AD-like lesions by repeated OX application. (**A**) Skin lesions observed in the three groups: control, AD, and PPE + AD group. (**B**) Skin thickness in the AD and PPE + AD groups was significantly higher than in the control group, denoted by *** (*p* ≤ 0.001), ** (*p* ≤ 0.01) and * (*p* ≤ 0.05), respectively. Notably, the skin thickness in the PPE + AD group showed a significant reduction compared to the AD group, indicated by ## (*p* ≤ 0.01) and # (*p* ≤ 0.05). The asterisk (*) indicates a significant difference between the AD and PPE+AD groups compared to the control group, while the hash (#) denotes a significant difference between the AD and PPE+AD groups. Statistical significance was assessed using the Mann–Whitney U-test. (**C**) Clinical scores were determined based on the severity of erythema, edema, itching, and excoriation of the skin. They were compared among the three groups at different time points, after 0, 7, 14, and 21 days of this study. The statistical analysis was performed using the Mann–Whitney U-test to compare the skin thickness and score among the three groups.

To explore the impact of OX on goat skin as a model for AD, we applied OX to a specific area of the abdominal skin in a time-dependent manner. Clinical evaluations were conducted every two or three days, focusing on erythema, edema, itching, and excoriation with hair loss of the skin. The application of OX on goat skin resulted in the development of AD-like lesions characterized by edematous erythema, accompanied by itching and dryness of the skin with crusts (Figure 2A). The clinical score, reflecting the severity of these lesions, was notably higher in the AD group compared to both the control and PPE + AD groups. Moreover, the skin thickness exhibited a significant reduction in the PPE + AD group (8.9 ± 1.3 mm, *p* < 0.05) compared to the AD group (22.8 ± 5.1 mm) (Figure 2B,C).

### 3.2. Pathological Assessment of AD Model in Goat

**Figure 3:**
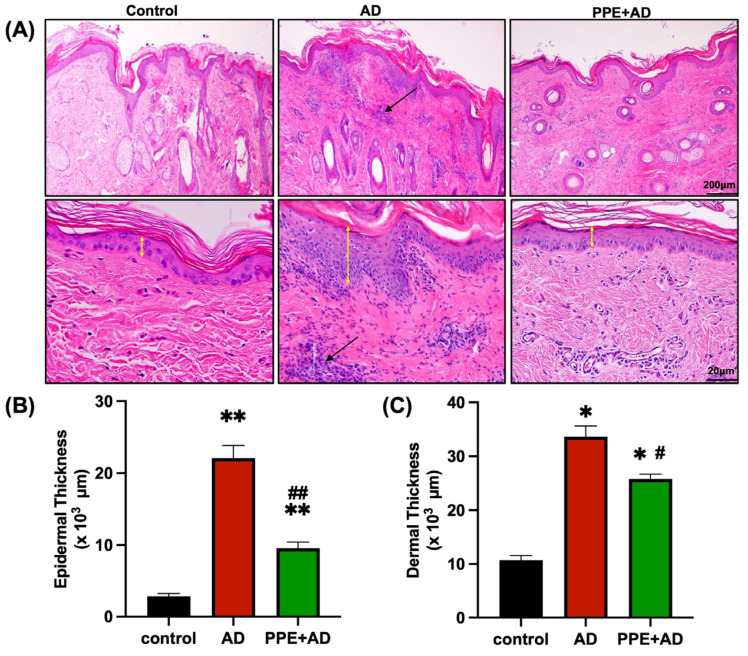
Histopathological analysis of goat skin. (**A**) Representative histopathological images depicting increased skin thickness and inflammatory cell infiltration (black arrows) in the three groups. (**B**,**C**) Significant elevation in epidermal and dermal thickness was observed in the AD group compared to both the control and PPE + AD groups (yellow arrows). Statistical significance in the graph was assessed using the Mann–Whitney U-test. A significant increase in epidermal and dermal thickness in the AD and PPE + AD groups compared to the control group is indicated by ** (*p* ≤ 0.01) and * (*p* ≤ 0.05), respectively. Comparisons between the AD and PPE + AD groups show significantly higher thickness in the AD group, denoted by ## (*p* ≤ 0.01) and # (*p* ≤ 0.05), respectively.

Histopathological features of skin biopsy in three groups are shown in Figure 3A. In the AD group, significant epidermal thickening and dermal thickening (22.08 ± 5.29 μm, *p* < 0.01; 33.61 ± 6.65 μm, *p* < 0.05, respectively) were observed compared to the control group (2.83 ± 1.21 μm; 10.7 ± 2.86 μm). The increased dermal thickness in the AD group was characterized by dermal edema and the presence of inflammatory cells, including mononuclear cells and granulocytes. The use of PPE in combination with OX resulted in a reduction in histopathological features, as evidenced by a reduction in cellular infiltration and a decrease in epidermal thickening and dermal thickening (9.55 ± 2.64 μm, *p* < 0.01; 25.78 ± 2.92 μm, *p* < 0.05) in the PPE + AD group compared to the AD group (Figure 3B). Pathological images for all animals per group were added to the supplementary file (Supplementary materials). 

**Figure 4:**
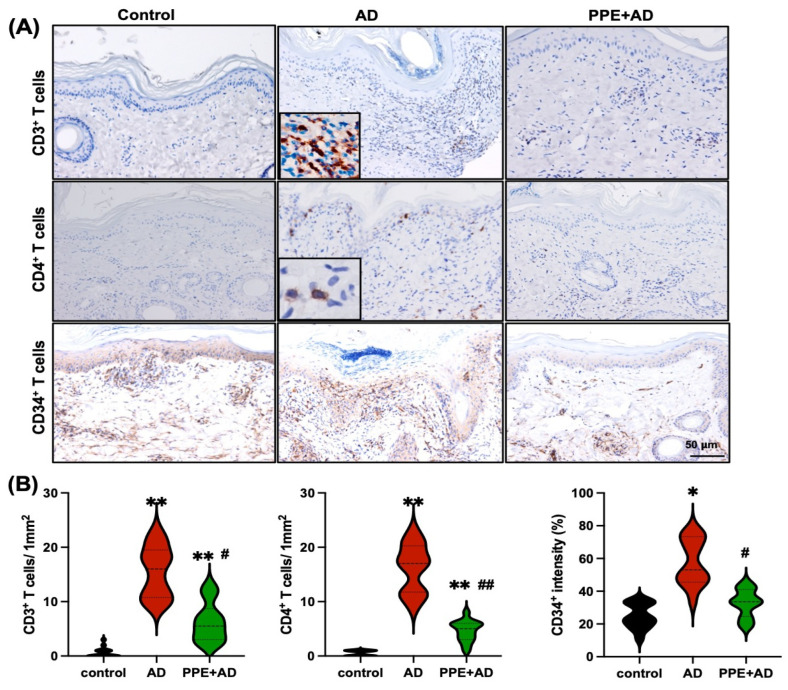
Immunohistochemical staining in goat skin tissue. (**A**) Representative images of immunohistochemical staining using monoclonal anti-CD3 and anti-CD4 antibodies in control, AD, and PPE + AD treated goats. (**B**) Comparative analysis of CD3^+^ and CD4^+^ T cells (/1 mm^2^) in the three groups using the Mann–Whitney U-test. The CD3^+^ and CD4^+^ in the AD and PPE + AD groups are significantly higher compared to the control group, indicated by ** (*p* ≤ 0.01), while CD34^+^ is significantly higher in the AD compared to the control group, indicated by * (*p* ≤ 0.05). Comparisons between the AD and PPE+AD groups reveal significantly higher expression of CD3^+^ and CD34^+^ in the AD group, indicated by # (*p* ≤ 0.05), and CD4^+^, indicated by ## (*p* ≤ 0.01).

To assess cellular infiltration, tissue sections were subjected to immunohistochemical staining using antibodies against CD3^+^, CD4^+^, and CD34^+^. The results revealed a higher frequency of CD3^+^ (15.5 ± 4.51 per 1 mm^2^) and CD4^+^ (11.57 ± 4.32 per 1 mm^2^) cells in the AD group compared to the control group (CD3^+^, 0.64 ± 0.89; CD4^+^, 0.5 ± 0.3 per 1 mm^2^) and the PPE + AD group (CD3^+^, 6.28 ± 3.49; CD4^+^, 4.46 ± 1.83 per 1 mm^2^), respectively. Additionally, the intensity of anti-CD34^+^ staining was significantly higher in the AD group (58.21%) compared to the other two groups, which showed values of 24.7% for the control group and 32.64% for the PPE + AD group, Figure 4A.

### 3.3. Molecular Assessment of AD Model in Goats

**Figure 5:**
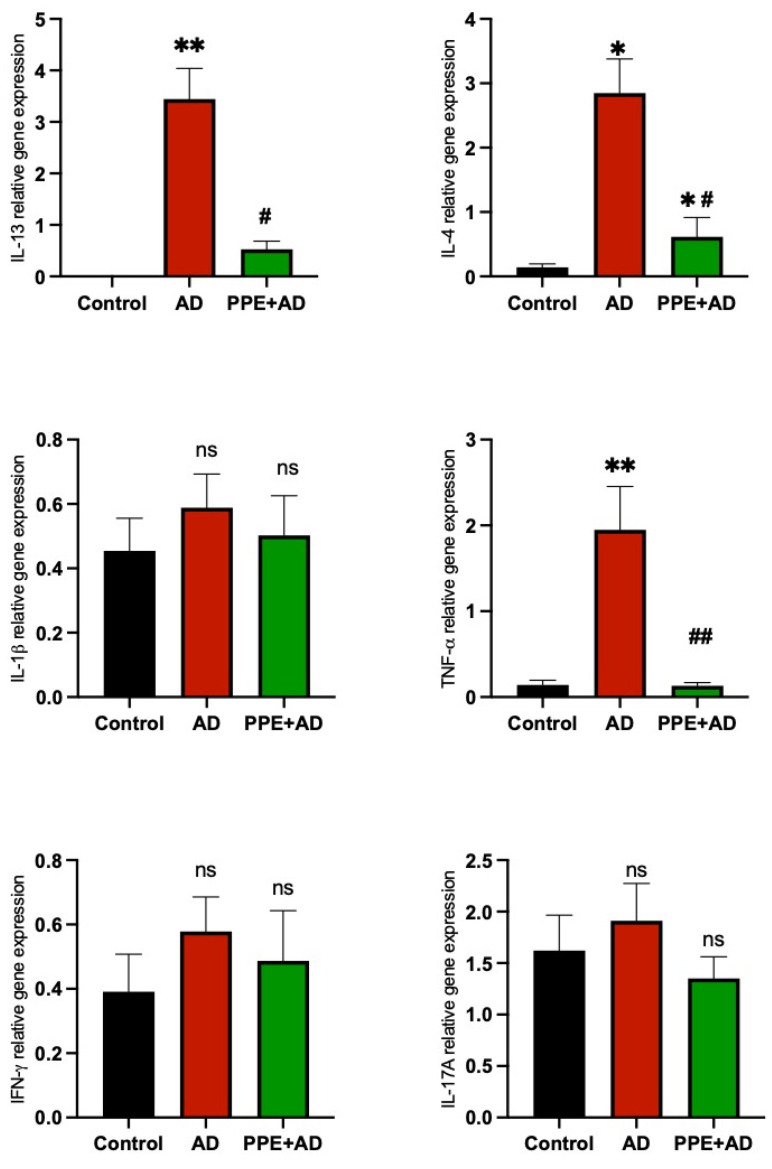
Expression of helper T (Th1) and Th2 cytokine genes in the goat model groups. Gene expression levels in the control, AD, and PPE + AD groups are presented as relative fold expression after normalization to two housekeeping genes. Bars and lines represent the mean and standard deviation (SD) (Mann–Whitney U-test). Statistical significance in the graph is indicated by ** (*p* ≤ 0.01) to show the significant increase in IL-13 and TNF-α in the AD group compared to the control group, while it is indicated by * (*p* ≤ 0.05) to indicate the significant increase in IL-4. Comparisons between the AD and PPE + AD groups show significantly higher IL-13 and IL-4 in the AD group, denoted by # (*p* ≤ 0.05), with a higher significance increase in TNF-α, denoted by ## (*p* ≤ 0.01).

To elucidate the immune response dynamics during the progression of atopic dermatitis in the goat model, alterations in tissue levels of Th1 cytokines (TNF-α, IFN-γ, and IL-1β), Th2 cytokines (IL-4, IL-13), and Th17 cytokines (IL-17A) were assessed. A significant increase was observed in the levels of IL-4 (*p* < 0.05) and IL-13 (*p* < 0.01) cytokines in the AD group compared to the control group. However, the levels of these same cytokines were significantly reduced in the PPE + AD group (*p* < 0.05) compared to the AD group. The Th1 cytokine TNF-α also showed a significant increase in the AD group compared to the other groups (*p* < 0.01). No significant (ns) differences were observed in IL-1β, IFN-γ, and IL-17A among the three groups (*p* > 0.05), Figure 5.

## 4. Discussion

Atopic dermatitis is prevalent among mammalian species, although with varying specific causes [19]. Therefore, it is crucial to identify animal models that closely resemble human and large animal physiology, to enhance our understanding of AD and formulate effective strategies for its treatment or prevention. This study demonstrates that in a goat model of AD, the skin undergoes morphological and molecular changes similar to those observed in mouse models. Given that essential AD features in goats align more closely with humans, this research underscores the promising utility of the goat model in advancing our knowledge of skin conditions.

Dermatitis models induced by repeated application of protein haptens, such as oxazolone on the skin of mice or dorsal ear, are commonly employed to simulate AD [20,21]. In this study, we selected goats as an alternative model to explore immune responses across different species. The clinical severity of AD was assessed through quantitative evaluations of skin symptoms, encompassing erythema, edema, itching with hair loss, and skin excoriation with crust formation (skin thickening). Pathological changes were positively correlated with clinical scores, contributing to an increase in both epidermal and dermal thickening in the AD model. Inflammatory cell infiltration, characterized by mononuclear cell infiltration, was also observed.

To confirm the induction of Th2-mediated AD lesions in the goat model through oxazolone application, we investigated inflammatory cell infiltration and cytokine expression in the skin tissue of both the control and AD groups. Real-time qRT-PCR results revealed a prevalence of Th2 cytokines over Th1 cytokines, with significantly higher transcription levels of IL-4 and IL-13 in the AD group compared to the control group. The heightened Th2 cytokine expression was correlated with the recruitment of CD3^+^ and CD4^+^ T cells to the oxazolone-treated tissue, indicating the presence of Th2-dominant inflammation. Additionally, CD34 intensity was evaluated as a marker of vascular endothelial activation and proliferation in the skin tissue. CD34 is a transmembrane glycoprotein commonly expressed in endothelial cells and associated with angiogenesis and tissue remodeling, particularly under inflammatory conditions. Although CD34 expression has been well documented in humans and other species, specific evidence in goats remains limited. In this study, the use of CD34 was based on the manufacturer’s predicted cross-reactivity with goat tissue and its relevance in similar models. The observed increase in CD34 expression suggests enhanced vascular activity and endothelial involvement in the inflammatory response [22,23]. This finding aligns with the histopathological evidence of inflammatory cell infiltration and increased dermal thickness in the AD group. The CD34 intensity in the PPE + AD group was comparatively lower, suggesting that PPE may have a regulatory effect on endothelial activation and vascular remodeling. Th17 cells have been explored as crucial players in AD in mouse models [5,7]. However, no significant difference in IL-17A cytokine expression was observed between the AD group and the control group in the goat model of our study. Further investigation into the role of Th17 cells in AD is warranted to better understand their involvement in the pathogenesis of this condition in different species.

Natural products serve as a valuable reservoir of medicinal agents, with over 50% of the most-prescribed drugs in the U.S.A. originating from natural sources [24]. Substances of natural origin have been extensively utilized in dermatology for their therapeutic efficacy in addressing skin issues, including anti-inflammatory, antimicrobial, and cell-stimulating properties [25]. In our efforts to introduce new anti-inflammatory materials from medicinal plants, we turned to pomegranate peel extract (PPE), renowned for its anti-inflammatory and antioxidant effects. The pomegranate peel is an excellent source of polyphenols, dietary fiber, vitamins, and other bioactive compounds. Utilizing byproducts from pomegranate processing, which is rich in these beneficial compounds, holds significant potential for developing a variety of products with antioxidant and anti-inflammatory properties [26]. PPE is rich in phytochemicals, including tannins, steroids, phenolics, alkaloids, flavonoids, terpenoids, and saponins. PPE exhibits potent anti-inflammatory effects by targeting key mediators of the inflammatory response. It effectively suppresses the expression of cyclooxygenase-2 (COX-2) and inducible nitric oxide synthase (iNOS), both of which play crucial roles in the inflammatory process. Additionally, PPE reduces the levels of pro-inflammatory cytokines such as TNF-alpha and IL-1 mRNA, further demonstrating its potential as a natural therapeutic agent for managing inflammation [27].

In this study, PPE exhibited a protective anti-inflammatory effect against the impact of oxazolone application on goat skin tissue. The pre-application of PPE mitigated the effects of oxazolone, as evident in the PPE + AD group compared to the AD group. Given that the levels of cytokines, including IL-4 and IL-13, are significant biological markers of allergic diseases like AD, our findings indicate that the pre-application of PPE led to a reduction in cytokine release and an improvement in the atopic damage observed in the goat AD model. Overall, the utilization of pomegranate peel extract as a therapeutic agent warrants further investigation and exploration. Our findings in the goat AD model demonstrate a Th2-dominant immune response, with elevated IL-4 and IL-13 cytokine expression, which closely mirrors observations in human and murine models of atopic dermatitis. In other species, AD is often associated with an overactive Th2 response, marked by increased levels of IL-4, IL-13, and IgE production, leading to inflammation, itching, and skin barrier disruption. Studies in murine models have also shown similar cytokine profiles, confirming the involvement of Th2 cells in driving AD pathology. Our goat model aligns well with these profiles, suggesting that goats may serve as a reliable large-animal model for studying the mechanisms of AD and testing new treatments. However, the use of goats as a model for studying atopic dermatitis (AD) in both humans and/or large animals presents itself as a reliable and accessible approach, but it still needs more investigation.

One limitation of our study is the validity of the antibodies used for immunohistochemical staining. While we selected antibodies predicted to work with ruminant tissues based on manufacturer guidelines, their specific reactivity with goat tissues remains uncertain, and further validation is necessary to confirm their effectiveness. However, while the goat model demonstrates the potential of PPE as a therapeutic agent for treating AD, further studies are required to validate its efficacy at different therapeutic doses. Additionally, we acknowledge the importance of incorporating other tools to validate the AD model, such as assessing skin barrier integrity through TEWL (transepidermal water loss) measurements or evaluating barrier proteins like filaggrin, loricrin, or claudin-1.

## 5. Conclusions

This preliminary study demonstrated that the topical application of oxazolone in the goat skin model successfully elicited a Th2-driven immune response, evidenced by the significant expression of Th2 cytokines (IL-4 and IL-13). These findings support the suitability of the goat model for studying atopic dermatitis (AD), offering closer physiological relevance to larger animals and humans compared to traditional murine models. Additionally, our results indicate that pomegranate peel extract (PPE) holds promise as a natural therapeutic agent for managing AD. However, further validation studies are required to confirm these findings, including the determination of optimal therapeutic doses, comparison with established treatments, and additional testing of the goat model’s robustness for AD research.

## Figures and Tables

**Table 1:** List of antibodies used in our study.

Antibody	Host and Type	Dilution	Source
anti-CD3	Primary, Rabbit monoclonal	1:150	Abcam, Cambridge, UK #clone SP7 ab16669
anti-CD4	Primary, Rabbit monoclonal	1:1000	Abcam EPR19514
anti-CD34	Primary, Rabbit polyclonal	1:200	Abcam EP373Y
IgG	Secondary, goat, HRP-conjugated	1:200	Abcam ab6721

**Table 2:** List of Th1, Th2, and Th17 cytokines primers used in our study.

Primer	Forward	Reverse	Reference
IL-17A	TTCTCTTTCCCAGGGCTACC	TCAACAAGATAATTGCTAATCTGACT	This study
TNF-α	CCTTGAGAAGATCTCACCTA	CAAACATAAACAGAGGGAGT	[17]
IL-1β	TACCTGTCTTGTGTGAAAAA	CAAATTCAACTGTGTTCTTG	[17]
IFN-α	GAGGAAATACTTCCACAGA	ATGACTTCTGCTCTGACAAC	[17]
IL-13	GTGTGGAGCCTCAACCTGAC	ACAGATGTGGGACTGAATGC	This study
IL-4	CAGCATGGAGCTGCCT	ACAGAACAGGTCTTGCTTGC	[18]
GAPDH	ATCTCGCTCCTGGAAGATG	TCGGAGTGAACGGATTCG	[18]
β-actin	TGGGCATGGAATCCTG	GGCGCGATGATCTTGAT	[18]

## Data Availability

The original contributions presented of this study are included in this article; raw data are available from the corresponding author upon reasonable request.

## Supplementary Materials

The following supporting information can be downloaded at: https://www.mdpi.com/article/10.3390/ani15030411/s1, Figure S1: Histopathology of all animals per group; Figure S2: Immunohistochemical staining of CD3^+^ for all animals per group; Figure S3: Immunohistochemical staining of CD4^+^ for all animals per group; Figure S4: Immunohistochemical staining of CD34^+^ for all animals per group.
