# Supplementary material for: Oxazolone-Induced Immune Response in Atopic Dermatitis Using a Goat Model and Exploration of the Therapeutic Potential of Pomegranate Peel Extract
Source: Animals (Basel). 2025 Feb 2;15(3):411. doi: 10.3390/ani15030411 (PMC11815913; doi:10.3390/ani15030411)
Supplement: Supplementary file 1 [file animals-15-00411-s001.zip › animals-3385660-supplementary.pdf]

<u>Title:</u> Oxazolone- induced immune response in Atopic Dermatitis Using a Goat Model and Exploration of the Therapeutic Potential of Pomegranate Peel Extract

<u>Pathological Assessment of AD Model in Goat</u>

Figure S1: Histopathology of all animals per group.

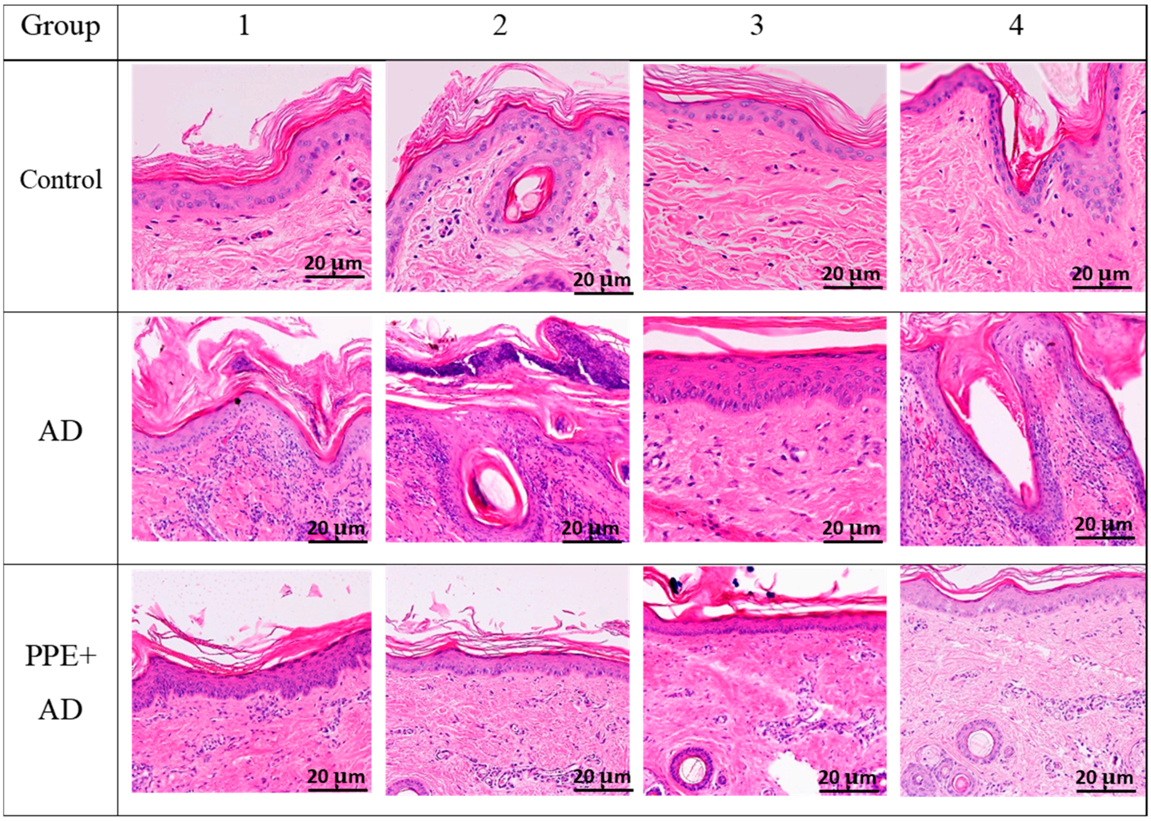

Figure S2: Immunohistochemical staining of CD3+ for all animals per group

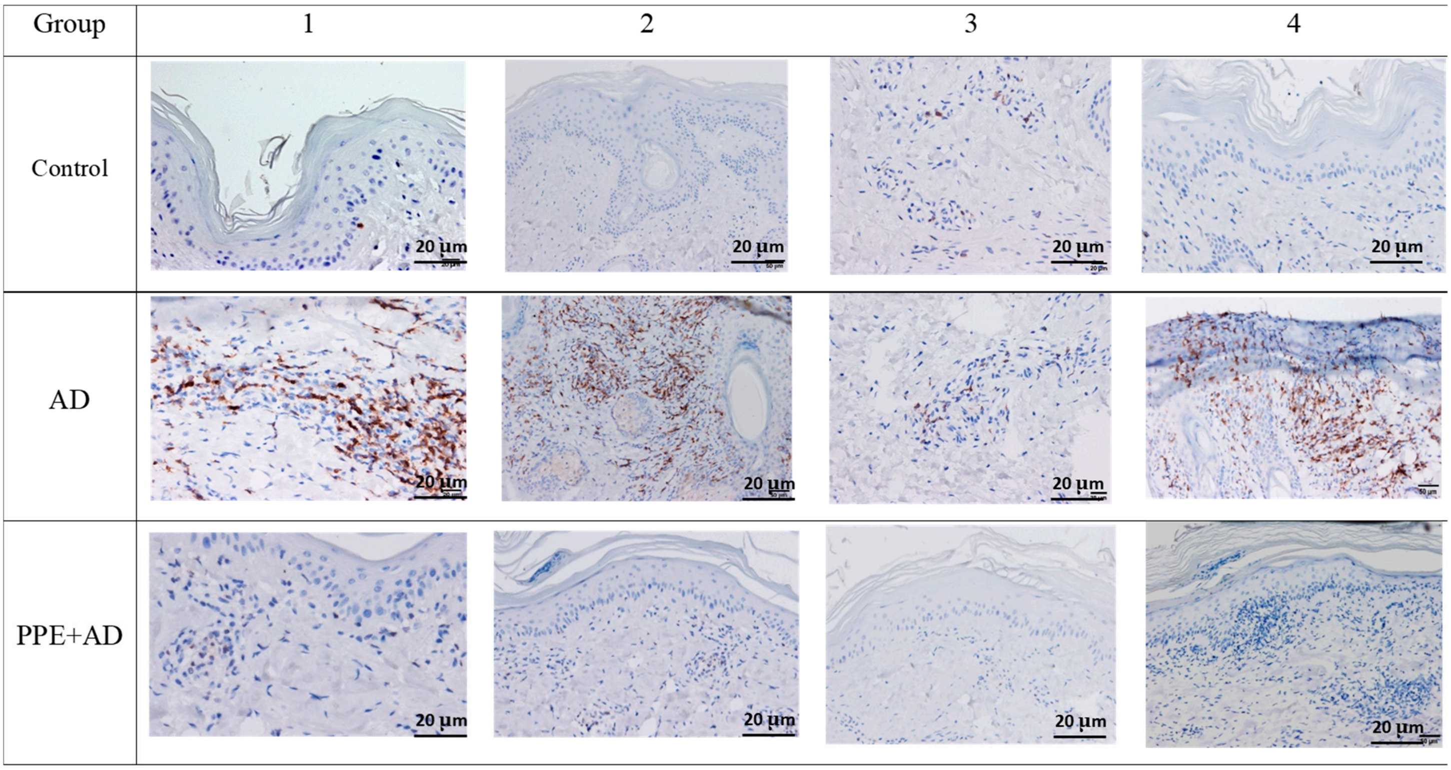

Figure S3: Immunohistochemical staining of CD4+ for all animals per group

Group	1	2	3	4
Control	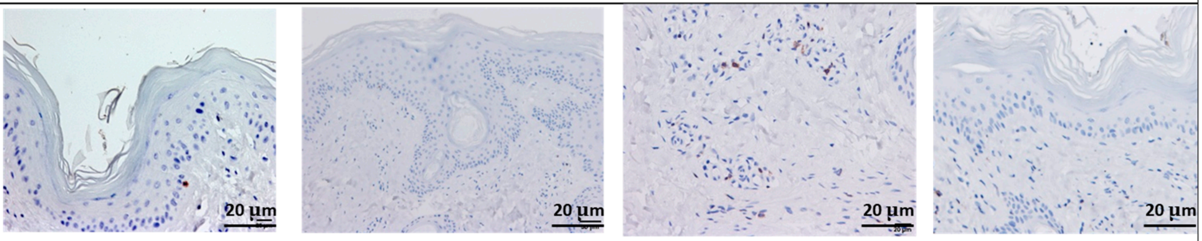			
AD	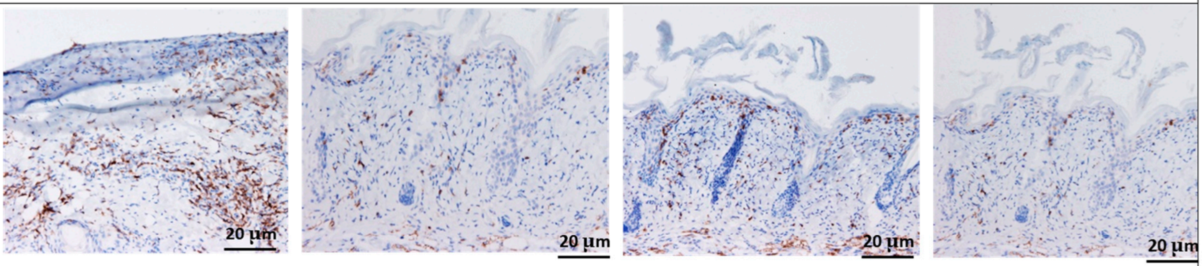			
PPE+ AD	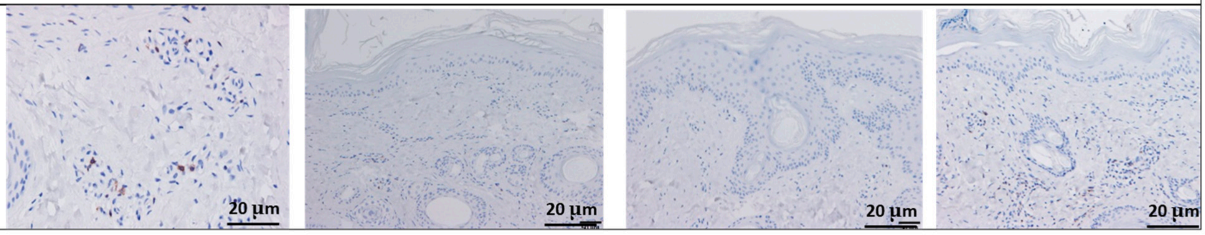			

Figure S4: Immunohistochemical staining of CD34+ for all animals per group

Group	1	2	3	4
Control	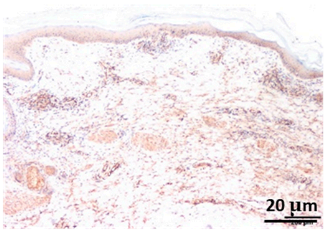	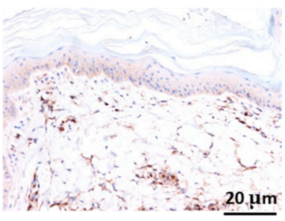	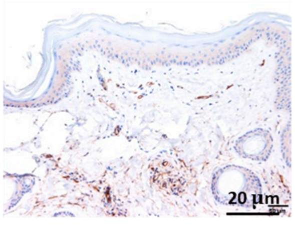	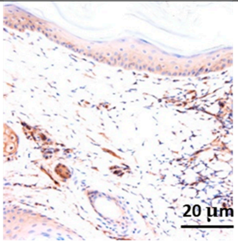
AD	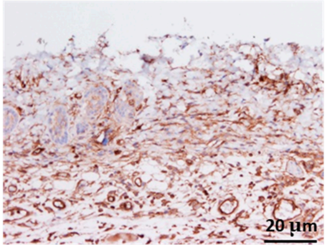	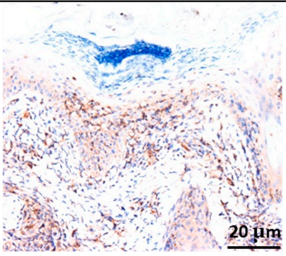	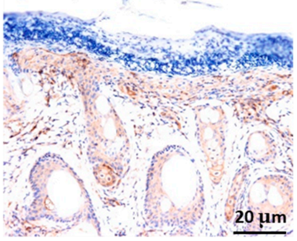	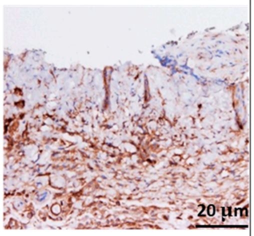
PPE+ AD	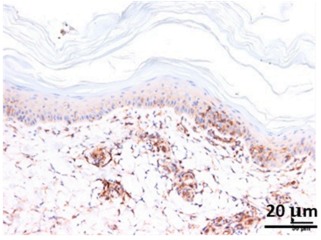	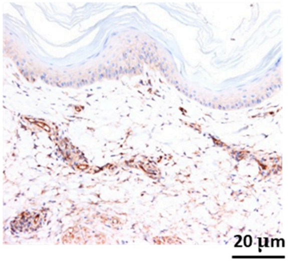	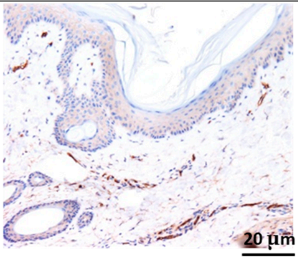	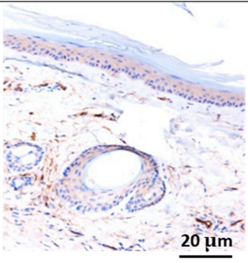
